# SIMA: Python software for analysis of dynamic fluorescence imaging data

**DOI:** 10.3389/fninf.2014.00080

**Published:** 2014-09-23

**Authors:** Patrick Kaifosh, Jeffrey D. Zaremba, Nathan B. Danielson, Attila Losonczy

**Affiliations:** Department of Neuroscience, Columbia University in the City of New YorkNew York, NY, USA

**Keywords:** calcium imaging, fluorescence imaging, *in vivo* GECI imaging, multi-photon microscopy, motion correction, Python language, analysis software, segmentation

## Abstract

Fluorescence imaging is a powerful method for monitoring dynamic signals in the nervous system. However, analysis of dynamic fluorescence imaging data remains burdensome, in part due to the shortage of available software tools. To address this need, we have developed SIMA, an open source Python package that facilitates common analysis tasks related to fluorescence imaging. Functionality of this package includes correction of motion artifacts occurring during *in vivo* imaging with laser-scanning microscopy, segmentation of imaged fields into regions of interest (ROIs), and extraction of signals from the segmented ROIs. We have also developed a graphical user interface (GUI) for manual editing of the automatically segmented ROIs and automated registration of ROIs across multiple imaging datasets. This software has been designed with flexibility in mind to allow for future extension with different analysis methods and potential integration with other packages. Software, documentation, and source code for the SIMA package and ROI Buddy GUI are freely available at http://www.losonczylab.org/sima/.

## 1. Introduction

Two-photon fluorescence imaging of neuronal populations has proven to be a powerful method for studying dynamic signals in neural circuits. For example, imaging of genetically-encoded fluorescent Ca^2+^ indicators (Looger and Griesbeck, 2012) has been widely applied to simultaneously monitor the activity in large populations of spatially, morphologically, or genetically identified neurons. These methods can be implemented *in vivo* in awake rodents (Dombeck et al., 2007; Komiyama et al., 2010; Lovett-Barron et al., 2014), providing the potential to study the molecular, anatomical, and functional properties of neurons responsible for behavior (Kerr and Denk, 2008; O'Connor et al., 2010). Relative to the electrophysiological approaches traditionally used to study neuronal activity *in vivo*, two-photon imaging provides the advantages of recording activity in entire local populations without spike-sorting ambiguities or bias toward highly active neurons, imaging activity in subcellular compartments such as axons or dendrites, and tracking the same neurons across experiments spanning multiple days. Additionally, fluorescence imaging can be used to measure other signals, such as membrane potentials and neurotransmitter release (Looger and Griesbeck, 2012).

To facilitate the analysis of data from dynamic fluorescence imaging experiments, we have developed two software tools: the Sequential IMaging Analysis (SIMA) Python package, and the ROI Buddy graphical user interface (GUI). The SIMA package can be used for motion correction, automated segmentation, and signal extraction from fluorescence imaging datasets. The ROI Buddy GUI allows for editing and annotating ROIs within a given imaging session, as well as registering ROIs across imaging sessions acquired at different times. The output data resulting from analysis with SIMA can either be directly analyzed using the NumPy/SciPy tools for scientific computing (Jones et al., 2001; Oliphant, 2007), or can be exported to common formats allowing for subsequent analysis with other software. The SIMA package and ROI Buddy GUI can be run on Linux, Windows, and MacOS operating systems, have been made freely available under an open source license, and require only other freely available open source software.

This manuscript provides an overview of the SIMA package and ROI Buddy GUI. Section 2 explains the capabilities of these software tools and how they can be used. Section 3 explains details of the algorithms that have been implemented to provide this functionality. Finally, Section 4 compares this software with other available resources and discusses potential future developments.

## 2. Functionality

**Figure 1:**
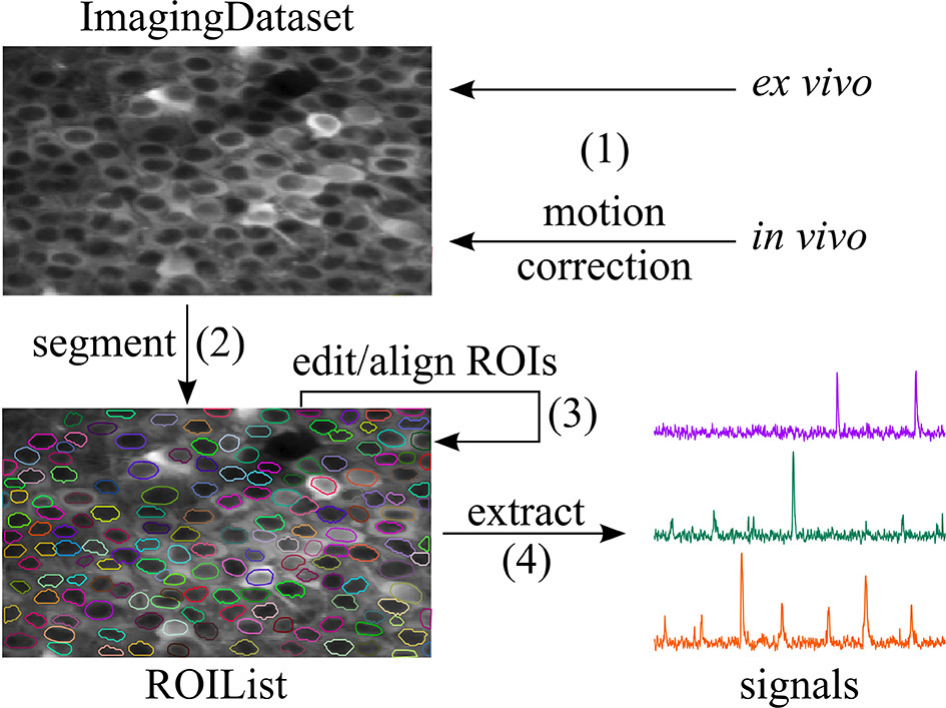
**Workflow supported by SIMA**. (1) An ImagingDataset object is first created either directly from the raw data or from the output of the motion correction algorithm. (2) ROIs are generated by automatic segmentation. (3) The ROI Buddy GUI can be used to edit the automatically generated ROIs and to automatically register ROIs across multiple datasets. (4) Dynamic fluorescence signals are extracted from the imaging data and ROIs.

The SIMA package and ROI Buddy GUI provide a variety of functionality outlined in Figure 1. To give an overview of this functionality, we provide sample code for typical use in the case in which the raw imaging data is contained in two NumPy arrays named channel_A and channel_B, (other possibilities for input data formats are discussed in Section 2.1), and in which the output data is to be stored in the location /save/path.sima. Throughout our code examples, we assume that the SIMA package has been imported with the import sima Python command.

With just a few lines of code, the user can correct motion artifacts in the data, and then segment the resulting ImagingDataset object to identify ROIs:


dataset = sima.motion.hmm(
    [[channel_A, channel_B]],
    ’/save/path.sima’)
dataset.segment()


If the data lack motion artifacts (e.g., in the case of fluorescence imaging in *ex vivo* brain slices), the motion correction step can be replaced with direct initialization of an ImagingDataset object. The full set of commands in this case is an follows:


dataset = sima.ImagingDataset(
    [[channel_A, channel_B]],
    ’/save/path.sima’)
dataset.segment()


In either case, the result of these commands is an ImagingDataset object containing the raw or motion-corrected imaging data and the automatically generated ROIs. This object is permanently stored in the location /save/path.sima so that it can be reloaded at a later time.

Following automated segmentation, the generated ROIs can be manually edited with the ROI Buddy graphical user interface (GUI). This GUI can be used to delete erroneous ROIs, add missing ROIs, merge ROIs that have been incorrectly split, and adjust the shapes and positions of existing ROIs. The ROI Buddy GUI can also be used to register ROIs across multiple datasets acquired at different times, allowing for assessment of long-term changes in neural activity.

Once the ROIs have been edited and registered, the ImagingDataset object can be loaded in Python again, and then dynamic fluorescence signals can be extracted from the ROIs as follows:


dataset = sima.ImagingDataset.load(
    ’/save/path.sima’)
dataset.extract()


The extracted signals are permanently saved with the ImagingDataset object and can be accessed at any time with the command dataset.signals(). If further analysis is to be performed with external software, the signals can be exported using the command dataset.export_signals(’/export/path.csv’).

The remainder of this section contains more detailed discussion of each of the stages of this workflow. This discussion complements the API documentation that is available online at http://www.losonczylab.org/sima.

### 2.1. Object classes and input formats

The SIMA package follows an object-oriented design. The central object class around which the package is structured is the ImagingDataset. Objects of this class can be created either by direct initialization or as the output of the motion correction function call. Direct initialization of an ImagingDataset object requires two mandatory arguments: (1) the raw imaging data formatted according to the requirements discussed below, and (2) the path where the ImagingDataset object is to be saved. Names for the channels may be specified as an optional argument. Once created, ImagingDataset objects are automatically saved to the designated location and can be loaded at a later time with a call to the ImagingDataset.load method.

A single ImagingDataset object can contain imaging data from multiple simultaneously recorded optical channels, as well as from multiple *cycles* (i.e., continuous imaging epochs/trials) acquired at the same imaging location during the same imaging session. To allow for this flexibility, the raw imaging data used to initialize the ImagingDataset object must be packaged into a list of lists, whose first index runs over the cycles and whose second index runs over the channels. For example, if the raw data is stored in an object called data, then the element data[i][j] corresponds to the jth channel of the ith cycle.

The formatting requirements for each such element of the aforementioned list of lists are designed to allow for flexible use of SIMA with a variety of data formats. The sole requirement is that each element be specified as a Python iterable object satisfying the following properties: (1) the iterable may not be its own iterator, i.e., it should be able to spawn multiple iterators that can be iterated over independently; (2) each iterator spawned from the iterable must yield image frames in the form of two-dimensional NumPy arrays; and (3) the iterable must survive Python's pickling and unpickling methods for saving and loading objects.

A simple example of an object that satisfies these requirements is a three-dimensional NumPy array, with the first index corresponding to the frame, the second to the row, and the third to the column. Therefore, data in any format can be analyzed with SIMA following conversion to a NumPy array object. We have also implemented the sima.iterables.MultiPageTIFF object class for creating SIMA-compatible iterables from multi-page TIFF files, and the sima.iterables.HDF5 object class for creating iterables from HDF5 files. For example, a two-channel dataset can be initialized from TIFF files as follows:


from sima.iterables import MultiPageTIFF
iterables = [[
    MultiPageTIFF(’channel1.tif’),
    MultiPageTIFF(’channel2.tif’)
]]
dataset = sima.ImagingDataset(
    iterables, ’/save/path.sima’,
    channel_names=[’GCaMP’, ’tdTomato’])


Compared to converting data from TIFF or HDF5 files to NumPy arrays, use of these custom iterables is advantageous because there is no need to duplicate the data for separate storage in a second format. Furthermore, less data need be held in memory at any one time because the MultiPageTIFF or HDF5 iterables allow for imaging data to be loaded one frame at a time on an as-needed basis.

Importantly, the SIMA package has been designed to allow for flexible extension with additional custom iterable classes analogous to the MultiPageTIFF class. Such extensions can be developed to allow SIMA to use data from any required input format. Therefore, users wishing to use SIMA with other data formats have two options: (1) to convert the data to a format already supported such as a TIFF stack or NumPy array, or (2) to extend SIMA by creating a new iterable type to support the desired data format.

### 2.2. Motion correction

**Figure 2:**
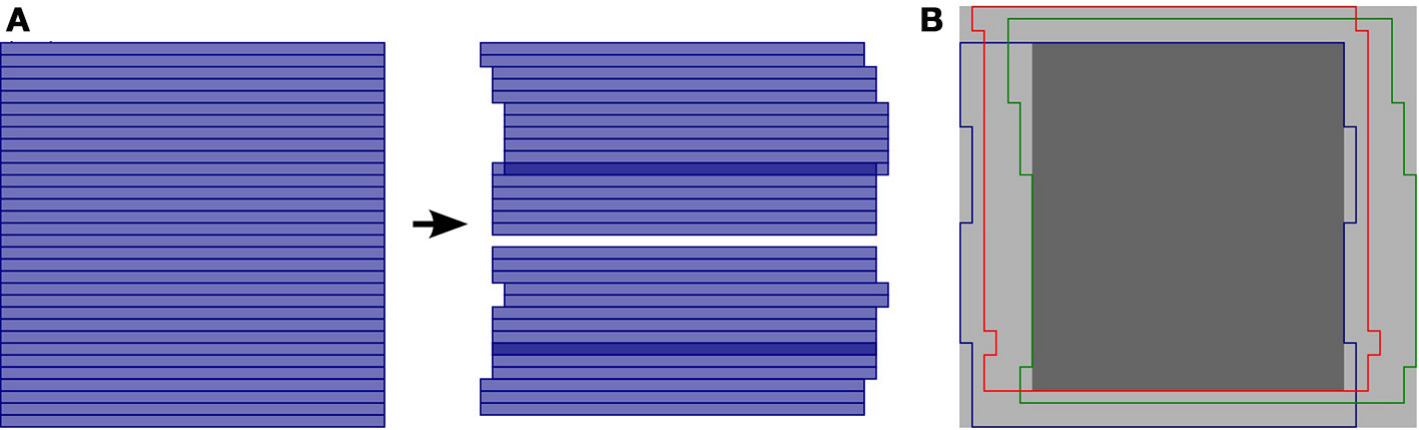
**Line-by-line correction of within-frame motion artifacts**. **(A)** Schematic diagram showing a single imaging frame before (left) and after (right) line-by-line motion correction. A separate displacement is calculated for each sequentially acquired line from the laser scanning process. As a result, some pixel locations may be accounted for multiple times (darker blue), while others may not be imaged in a given frame (white gap). **(B)** Overlay of different regions imaged by different frames due to motion. The light gray region indicates the maximum frame-size that can be selected for the motion correction output, such that all pixels locations that were ever imaged are within the frame. The dark gray region indicates the default and minimum frame-size that can be selected for the motion correction output, such that all pixels locations within the frame are within the field of view at all times.

During awake *in vivo* laser-scanning microscopy, the animal's movements cause time-dependent displacements of the imaged brain region relative to the microscope and thus introduce substantial artifacts into the imaging data. These artifacts are especially problematic when attempting to extract transient fluorescence signals from very small structures, such as dendritic branches and synaptic boutons (e.g., Kaifosh et al., 2013). Since individual pixels are acquired at different times during laser scanning microscopy, motion artifacts can occur within a single frame and cannot be corrected by simple frame alignment methods. To allow for correction of these within-frame motion artifacts, the SIMA package includes line-by-line motion correction software (Figure 2) that we developed (Kaifosh et al., 2013) by extending upon the hidden Markov model (HMM) approach used by Dombeck et al. (2007).

A call to the motion correction function sima.motion.hmm returns a motion-corrected ImagingDataset object. This function takes the same arguments used to directly initialize an ImagingDataset object, as well as additional arguments for specifying parameters for the motion correction procedure. One optional argument allows for specification of the number of states retained at each step of the Viterbi algorithm (see Section 3.1.1 for details). Retaining a larger number of states may in some cases result in more accurate displacement estimates, though at the expense of longer run-times. The maximum allowable displacement in the horizontal and vertical directions can also be specified. Use of this restriction can improve the quality of the estimated displacements by ruling out unreasonably large estimates. Optionally, a subset of the channels can be selected for use in estimating the displacements, which will then be used to correct artifacts in all channels. This option is useful in cases where there is a sparse or highly dynamic channel with signals of interest, and an additional static channel providing a stable reference for motion correction.

Once the motion artifacts are corrected, the frames of the resulting ImagingDataset show static imaged structures, but a field of view that moves from frame to frame (Figure 2B). Typically, a frame size larger than that of the original images is required to display the full spatial extent that was imaged during the session. Relatedly, the area imaged during all frames is smaller than that of the original images. To determine the spatial extent of the corrected image series that will be retained for further analysis, the hmm function takes an additional optional argument, the trim_criterion, which specifies the fraction of frames for which a location must be within the field of view in order to be retained for further analysis. By default, the edges of the corrected images are conservatively trimmed to retain only the rectangular region that remains within the field of view during all imaging frames.

### 2.3. Segmentation and ROIs

The SIMA package allows for automated segmentation with a call to the ImagingDataset.segment method. The segment method takes arguments that allow for specification of the approach to be used and an optional label for the resulting set of ROIs, which are saved with the ImagingDataset. Arguments specific to the particular method can also be passed into this method call. The SIMA package currently contains two implemented segmentation methods, ’normcut’ and ’ca1pc’, both of which are based on the normalized cuts approach (Shi and Malik, 2000). Further details on these particular segmentation approaches are provided in Section 3.2.

A call to the segment method returns an ROIList object, which contains the segmented ROI objects. As well, ROI objects can be initialized independently in one of four ways: (1) with a mask, typically a NumPy array, indicating the weight of each pixel (see Section 3.4), (2) with a list of polygons, each consisting of a list of vertices, (3) using ROI Buddy (see Section 2.4), or (4) by importing a set of ROIs created in ImageJ (Schneider et al., 2012). Masks can either be binary, to select a subset of pixels, or real-valued, as in the case of weights resulting from principal or independent component analysis. Polygons are treated equivalently to binary masks. ROIs typically consist of a single polygon, however multiple polygons are useful for marking structures that leave and re-enter the imaging plane.

Additionally ROI objects have the following optional attributes: id, label, and tags. The label attribute is a descriptor for the ROI used for referencing the region within one imaging session. The id of an ROI object is an identifier used to track the region over multiple imaging sessions, such that two ROI objects from different experiments that have the same id are understood to correspond to the same neuron/dendrite/bouton. The id values are automatically set during ROI registration with the ROI Buddy GUI. The tags attribute is a set of strings associated with the ROI, used for sorting or marking the ROIs based on morphological, genetic, or other criteria. These tags can also be modified from within the ROI Buddy GUI or during analysis of fluorescence signals to aid in the selection and sorting of ROIs during subsequent analysis.

### 2.4. Manual ROI editing

**Figure 3:**
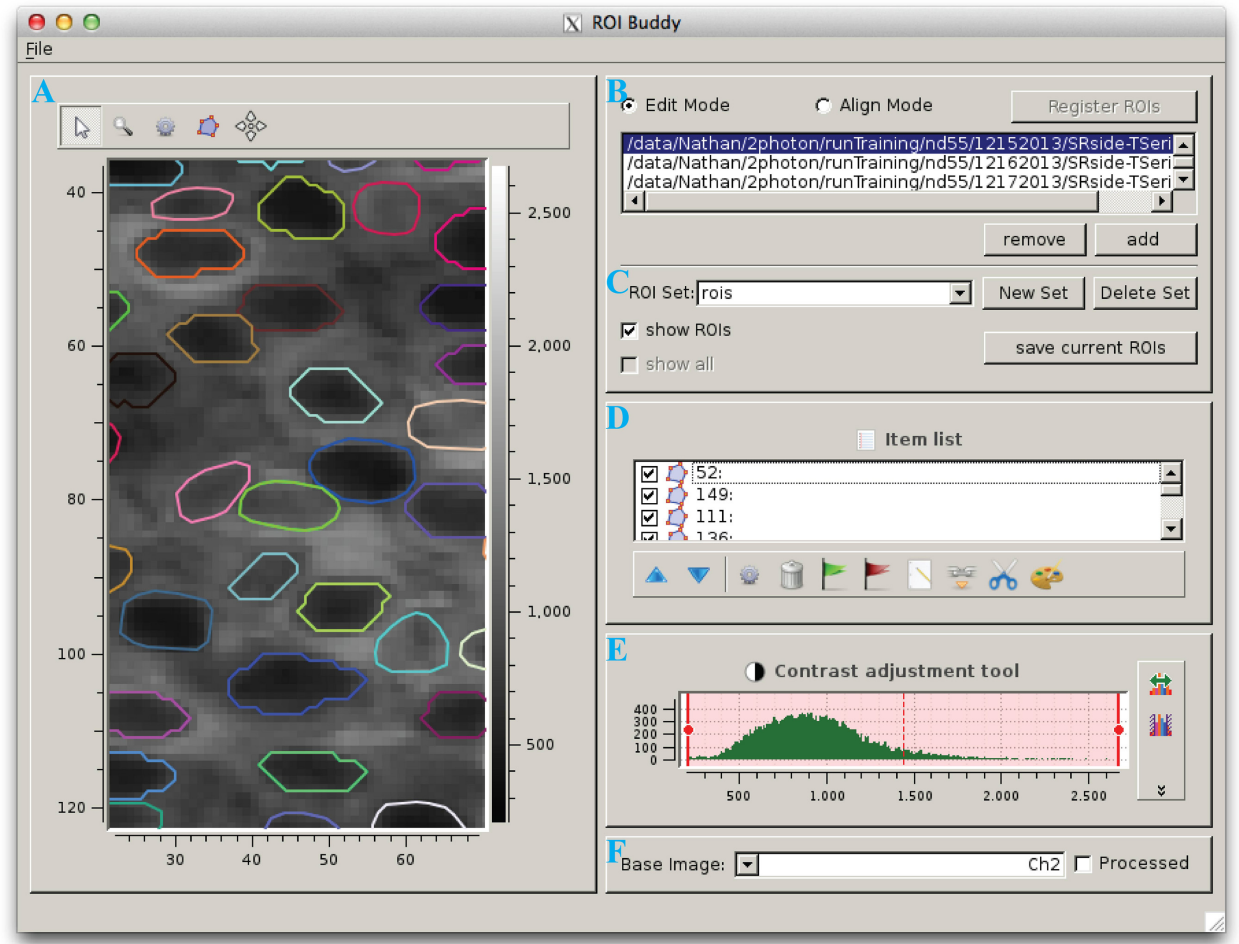
**The ROI Buddy graphical user interface**. **(A)** Image viewing panel with ROI editing tools. During typical use this panel is expanded to occupy the majority of the screen. **(B)** Panel for toggling between “Edit” and “Align” modes, loading imaging datasets, and registering ROIs across datasets. **(C)** Panel for selecting, creating, saving, and deleting sets of ROIs associated with the active imaging dataset. In “Align” mode, ROIs from all loaded datasets can be viewed simultaneously. **(D)** List of ROIs in the currently selected set, and tools for tagging, merging, unmerging, and re-coloring ROIs. **(E)** Contrast adjustment for the underlying base image. **(F)** Panel for selection of the underlying base image.

The ROI Buddy GUI can be used to view and edit the automated segmentation results or to manually draw new ROIs (Figure 3). When the user loads an ImagingDataset object, the time-averaged images are displayed as a static background on which ROI objects are displayed. The underlying static image can be toggled between each of the imaged channels, and optionally a contrast-enhanced “processed” image can be displayed. Each ROI object, consisting of one or more polygons, is displayed with a unique color over this background. If multiple ROIList objects are associated with an ImagingDataset (automatically generated and manually edited sets, for example), the active set is selectable via a drop-down menu. The user can also toggle between simultaneously loaded ImagingDataset objects, which is useful for rapidly switching between multiple imaging sessions of the same field of view in order to verify the ROIs during editing.

Once the ImagingDataset and ROI objects are loaded in the GUI, the user can edit, delete, and add new ROIs as polygons while in the GUI's “Edit” mode. All ROIs are directly editable, allowing for the user to adjust individual vertices or translate the entire ROI. In addition, separate polygons can be merged either into a single multiple-polygon ROI or, if the polygons are overlapping, into a single polygon ROI. The interface also allows the user to directly set the label and tags properties of each ROI described in Section 2.3.

### 2.5. ROI registration

**Figure 4:**
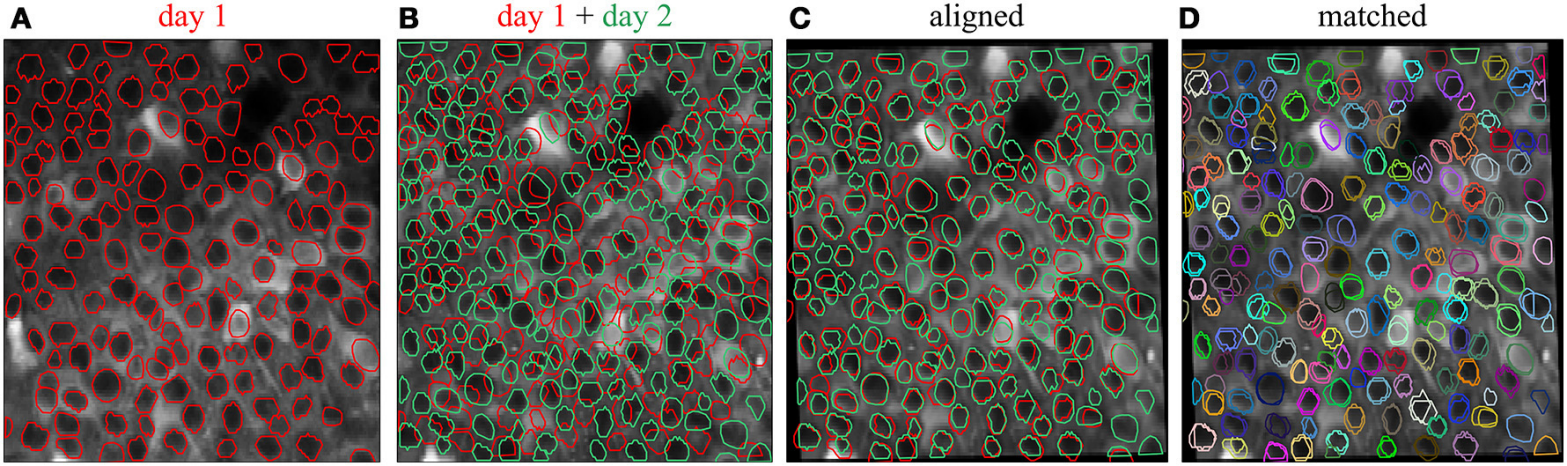
**Registration of ROIs across imaging sessions acquired on two different days**. **(A)** ROIs (red) and time-averaged image for the first imaging session. **(B)** ROIs (green) and time-averaged image for the second imaging session, with ROIs for the first imaging session (red) shown for comparison. **(C)** Same as **(B)** but with an affine transformation applied to align the time-averaged image and ROIs from day 2 to those of day 1. **(D)** Same as **(C)** but with the ROIs colored by their automatically determined shared identities across both imaging sessions.

To track the same structures over multiple imaging sessions of the same field of view (Figure 4), the ROI Buddy GUI also supports the registration of ROIs from different ImagingDataset objects. In the GUI's “Align” mode, affine transformations are estimated to align the time-averaged images of the currently active ImagingDataset with each of the other loaded sets. These transformations are then applied to the respective ROI objects to transform them all into the space of the active ImagingDataset (Figure 4C). This allows ROIs to be imported from one set on to the active ImagingDataset or for all of the ROIs to be viewed simultaneously over the time-averaged image of a single ImagingDataset. The ROIs are then automatically identified across imaging datasets based on their degree of overlap following transformation. The id attributes of co-registered ROI objects are set to be equal, thus allowing for tracking of the same regions over multiple imaging sessions.

When displayed in the GUI, co-registered ROI objects are colored identically for easy visual inspection of the registration results (Figure 4D). Groups of co-registered ROIs can be manually modified by removing and adding ROI objects to correct any errors in the automated registration. The tags can also be propagated across co-registered ROIs from different ImagingDataset objects.

### 2.6. Signal extraction

Signal extraction is accomplished by the ImagingDataset.extract method. This extract method can take several optional arguments. The ROIList to be used can be specified in cases where there are multiple ROIList objects (e.g., one that has an automatically generated and another that has been manually edited) associated with the ImagingDataset. If multiple optical channels are present, the channel to be used for extraction can be specified. If the ROIs are either polygons or a binary masks, the extract method can optionally exclude pixels that overlap between ROIs in order to reduce artifactual correlations between adjacent ROIs.

The output of the extract method is a Python dictionary, which is also automatically saved as part of the ImagingDataset object. This dictionary contains (1) the raw extracted signals, (2) a time-averaged image of the extracted channel, (3) a list of the overlapping pixels, (4) a record of which ROIList and channel were used for extraction, and (5) a timestamp. Additionally, a verification image is saved as a PDF file showing the extracted ROIs and overlapping pixels overlaid on the time-averaged image. Once the signals are extracted, they can be accessed at any time with a call to the ImagingDataset.signals method.

### 2.7. Exporting data

The SIMA package is intended to provide support for early stages of data analysis, such that subsequent analysis of the extracted signals can be performed with separate software. In cases where all analysis is performed using Python, no exporting is necessary, since the SIMA objects can be used in conjunction with other Python code. In other cases, data from SIMA objects can be easily exported into standard formats, including TIFF images and CSV text files.

Such exporting of data can be performed at various stages of data processing with the SIMA package. For example, those wishing to use SIMA solely for motion correction can export the motion-corrected time series with a call to the ImagingDataset.export_frames method. This method takes as its argument the filenames with which the exported data will be saved, formatted as a list of lists of strings organized similarly to the input data (see Section 2.1). Additional optional arguments can be used to specify the output file format, whether to scale the intensity values to the full range allowed by the output file format, and whether to fill in unobserved rows (Figure 2A) of motion corrected images with values from adjacent frames. Time-averaged images can similarly be exported with the ImagingDataset.export_averages method.

If SIMA is also used for signal extraction, then the extracted signals can be exported to a CSV file with the ImagingDataset.export_signals method. The resulting CSV file contains the id, label, and tags for each ROI, and the extracted signal from each ROI at each frame time.

## 3. Software details

### 3.1. Motion correction

We have previously described the HMM formulation and parameter estimation procedures that we have implemented for correction of within-plane motion during laser scanning microscopy (Kaifosh et al., 2013). Here we provide some additional details about the software implementation.

#### 3.1.1. Viterbi-based algorithm

The Viterbi algorithm computes the maximum *a posteriori* sequence of states for a HMM. For a general HMM with *S* hidden states and *T* timesteps, the Viterbi algorithm has time complexity *O*(*S*^2^*T*). When used for motion correction, the hidden states are the possible displacements, with one state per pair of *x* and *y* integer displacements in pixel units. By restricting state transitions to those between nearest neighbors in two dimensions, we reduce the complexity of the algorithm implemented in SIMA to *O*(*ST*). This restriction is justified by the same assumption—that negligible motion occurs during the time required to image a row—by which we justify applying the same displacement to all pixels in the same row. Some of the datasets from our laboratory exhibit substantial displacements in two dimensions, resulting in the number of states *S* being rather large; however, at any one time-step, the probability is typically concentrated in a much smaller number of states. Our software exploits this concentration of probability by retaining only the *N* ≪ *S* most probable states at each time-step. This approximation of the Viterbi algorithm reduces the computational complexity to *O*(*NT*).

Further increases in speed have been achieved by storing precomputed results for a number of transformations applied to the reference image and the image being aligned. These transformations include scaling by the estimated gain factor (see Kaifosh et al., 2013) to convert intensity values to estimated photon counts, and computation of the logarithm and gamma functions applied to these scaled values. Repeated computations have also been avoided by using lookup tables for the indices of overlapping pixels between the image and the reference frame, for the possible transitions between hidden states, and for the probabilities of the transitions.

### 3.2. Segmentation

**Figure 5:**
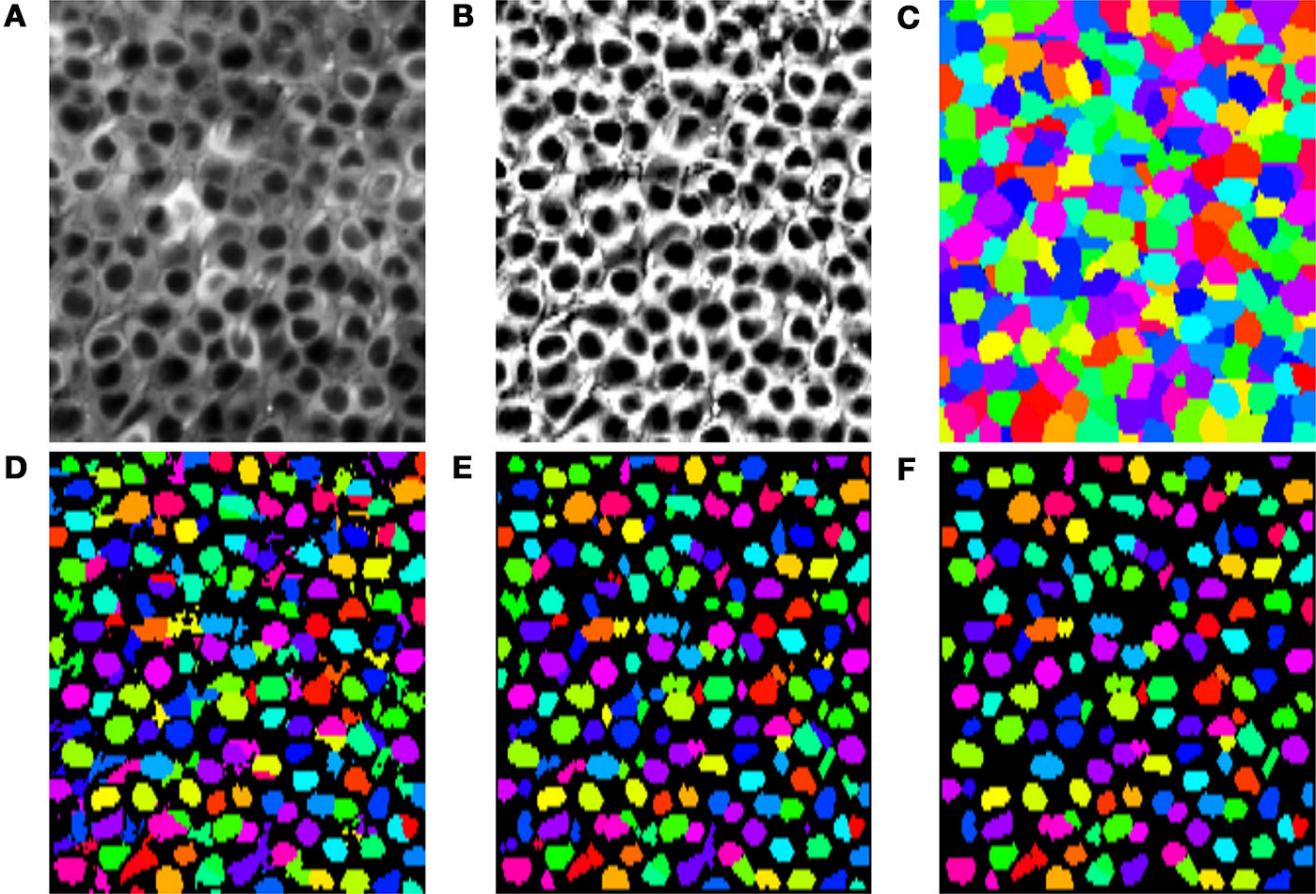
**Segmentation steps or identifying pyramidal cell nuclei with the ’ca1pc’ variant of the normalized cuts segmentation approach**. **(A)** The time-averaged image of the time-series to be segmented. **(B)** Application of CLAHE and unsharp mask image processing to **(A)**. **(C)** Disjoint regions identified by iterative partitioning with the normalized cuts algorithm. **(D)** Local Otsu thresholding of each region in **(C)**. **(E)** Cleanup of the Otsu thresholded regions in **(D)** with opening and closing binary morphology operations. **(F)** Resulting ROIs after rejection of regions in **(E)** that failed to satisfy minimum size and circularity requirements.

Although SIMA is designed to be extended to allow for multiple approaches to segmentation, the initial release includes only two segmentation methods, both using the normalized cuts approach (Shi and Malik, 2000). Specifically, we have implemented a basic normalized cuts segmentation (’normcut’), as well as a variant designed for segmentation of pyramidal cell nuclei in hippocampal area CA1 (’ca1pc’). Here, we describe first how we use the normalized cuts approach to partition the field of view, and then how, in the case of the ’ca1pc’ variant, these regions are post-processed to create ROIs (Figures 5A–F).

#### 3.2.1. Normalized cut formation

The normalized cut segmentation algorithm (Shi and Malik, 2000) partitions the imaged field of view through an iterative process. At each stage, a subset of the image pixels is split into two new subsets in such a way as to minimize a penalty that depends on a set of connection weights between the pixels. The resulting normalized cuts are uniquely determined by two factors: (1) the connection weights between pixels, and (2) the termination criterion for the iterative splitting procedure.

For the standard normalized cuts procedure implemented in SIMA, the weight *w*_*ij*_ between each pair of pixels *i* and *j* is calculated as follows:

(1) $$w_{ij}=e^{k_{c}c_{ij}}\cdot \{\begin{array}{ll} e^{-\frac{||x_{i}\;-\;x_{j}|{|}^{2}}{\sigma_{x}^{2}}} & \text{if }||x_{i}-x_{j}||<r\\ 0 & \text{otherwise} \end{array},$$

where *c*_*ij*_ is an estimate of the correlation between the pixels' intensity signals, ∥**x**_*i*_ − **x**_*j*_∥ is the Euclidean distance between the positions **x**_*i*_, **x**_*j*_ of the pixels, and σ^2^_**x**_ specifies the decay of weights with distance up to a maximum distance *r*. We set the parameter *k*_*c*_ = 9 based on empirical observations of segmentation accuracy.

For the ’ca1pc’ variant, we use a different set of weights *w*^CA1PC^_*ij*_, which are calculated by multiplying the weights *w*_*ij*_ from Equation (1) by a factor depending on the maximum pixel intensity along a line connecting the two pixels. Specifically, the modified weights are defined as

(2) $$w_{ij}^{\text{CA1PC}}=w_{ij}\cdot \exp\left(-k_{I}\underset{s\;\in \;[0,1]}{\max}I_{\text{avg}}^{*}\left((1-s)x_{i}+sx_{j}\right)\right),$$

where I^*^_avg_(**x**) is the intensity at location **x** of the time-averaged image, processed with Contrast Limited Adaptive Histogram Equalization (CLAHE) and an unsharp mask in order to correct intensity inhomogeneities and enhance the contrast (Figure 5B). Based on empirical observations of segmentation accuracy, we set *k*_*I*_ = 3/(max I^*^_avg_ − min I^*^_avg_), with the maximum and minimum taken over the entire image. The effect of this modification is to increase the weights between two pixels within the same low-intensity pyramidal cell nucleus relative to the weights between other pixels.

The termination criterion for the iterative partitioning of the image depends on the number of pixels in the region and the normalized cut penalty for the next potential partitioning. Specifically, partitions containing fewer than a minimum number of pixels (cut_min_size) do not undergo further partitioning, whereas partitions with greater than a maximum of pixels (cut_max_size) always undergo further partitioning. For partitions with an intermediate number of pixels, further partitioning occurs only if the penalty associated with the partitioning would be below a given threshold. For populations of uniformly sized neurons, such as those in the pyramidal layer of CA1, suitable termination is achieved when the values for cut_max_size and cut_min_size are chosen as upper and lower bounds on the typical cell size. An example set of partitions obtained with the ’ca1pc’ variant is shown in Figure 5C.

#### 3.2.2. Post-processing of partitions

In contrast to the basic ’normcut’ segmentation method, which simply returns the partitions as the ROIs, the ’ca1pc’ variant applies a series of post-processing steps to these partitions to isolate the darker pixels corresponding to the putative CA1 pyramidal cell nuclei. First a threshold is calculated for each partition based on Otsu's method (Otsu, 1979) for cluster-based thresholding, allowing for the rough separation of light and dark pixels (Figure 5D). Following this step a series of morphological operations, consisting of a binary opening followed by a binary closing, are applied to each identified region to regularize the ROI shapes by filling in gaps and smoothing the borders of each region (Figure 5E). Finally a minimum size and circularity criterion is applied to each region to reject small and irregularly shaped regions (Figure 5F).

We evaluated this ’ca1pc’ segmentation algorithm on two-photon fluorescence imaging data from GCaMP6f-expressing pyramidal cells in hippocampal area CA1 (see Lovett-Barron et al., 2014 for methodological details). Each of the 37 datasets consisted of 4575 frames of size 128 × 256 pixels acquired at 7.6 Hz with a 40× Nikon immersion objective at optical zoom 2×. We ran the segmentation algorithm with the following parameters: num_pcs=50, max_dist=(3, 6), spatial_decay=(3, 6), cut_max_pen=0.10, cut_min_size=50, cut_max_size=150, x_diameter=14, y_diameter=7, circularity_threhold=0.5, min_roi_size=20, min_cut_size=40. We compared the automatically segmented ROIs with manually curated segmentation. With a minimum Jaccard index of 0.25 as the criterion for a match between ROIs, the automatic segmentation had a false negative rate of 12 ± 2% and a false positive rate of 20 ± 5% (mean ± *SD*).

### 3.3. ROI registration

To estimate affine transformations between pairs of time-averaged images, we used the getAffineTransform function from OpenCV. Once ROIs are transformed into the same reference space, the ROI Buddy GUI can automatically estimate the degree of similarity between each pair of ROIs from different ImagingDataset objects by calculating the Jaccard index, defined as the area of the intersection divided by the area of the union. ROIs are then clustered with the unweighted pair group method with arithmetic mean (UPGMA) hierarchical clustering algorithm (Sokal and Michener, 1958), with distances between ROIs given by the reciprocal of the Jaccard index for that pair. ROI pairs from the same ImagingDataset are assigned infinite distance to prevent co-clustering of ROIs from the same imaging session. The termination criterion for clustering is set such that pairs of ROIs in a cluster have a minimum Jaccard index of 0.25. The objects of each cluster are then assigned a common id attribute, allowing for identification of the same region over multiple imaging sessions.

### 3.4. Signal extraction

In discussing the extraction procedures, we use the notation *w*_*ip*_ to denote the weighting of the *p*th pixel by the *i*th ROI. For polygon or binary mask ROIs, created with SIMA's automated segmentation or the ROI Buddy GUI, or imported from ImageJ, *w*_*ip*_ is defined as $\frac{1}{N_{i}}$ for pixels *p* within the ROI and 0 elsewhere, where *N*_*i*_ is the number of pixels in the *i*th ROI.

The simplest case for extraction occurs when the same pixel locations are imaged in every frame. In this case, we calculate the signal by a simple weighting of the normalized fluorescence intensities from each pixel. Specifically, the signal of the *ith* ROI at time *t* is calculated as

(3) $$s_{it}=\sum_{p}w_{ip}\cdot \frac{f_{pt}}{f_{p}},$$

with *f*_*pt*_ denoting the intensity of the *p*th pixel in the frame at time *t*, and *f*_*p*_ denoting the average intensity across all frames at pixel location *p*.

When extracting signals following correction of within-frame motion artifacts, the situation is complicated by the fact that not all pixel locations are observed in each frame. To derive a method for extracting these signals, we first note that the simple extraction method (Equation 3) reduces to the least-squares error estimate for a simple linear model in which the pixel intensities are related to the underlying ROI signals as follows:

$$\frac{f_{pt}-f_{p}}{f_{p}}=\sum_{i}a_{pi}(s_{it}-s_{i}^{*}),$$

with the coefficients *a*_*pi*_ defined as the entries of the pseudoinverse of the matrix with entries given by the weights *w*_*ip*_, and with s^*^_i_ set as ∑_*p*_ *w*_*ip*_. Given this model, when a subset *P*_*t*_ of the pixels are imaged in the frame taken at time *t*, the least squares estimate of the signal is given by

$$s_{it}=\sum_{p}w_{ipt}\cdot \frac{f_{pt}-f_{p}}{f_{p}}+\sum_{p}w_{ip}.$$

Here, the time-dependent coefficients *w*_*ipt*_ are defined as the entries of the pseudo-inverse of the matrix with entries *a*_*pi*_ for all pixels *p* in *P*_*t*_.

A few special cases are worth mentioning. For non-overlapping ROIs, this formula reduces to

$$s_{it}=\frac{\sum_{p}w_{ip}^{2}}{\sum_{p\;\in \;P_{t}}w_{ip}^{2}}\cdot \sum_{p\;\in \;P_{t}}w_{ip}\frac{f_{pt}-f_{p}}{f_{p}}+\sum_{p}w_{ip}.$$

In cases of binary mask or polygon ROIs, the above formula simplifies to

$$s_{it}=\frac{1}{N_{it}}\cdot \sum_{p\;\in \;P_{it}}\frac{f_{pt}}{f_{p}},$$

where *P*_*it*_ is the set of pixels in the *i*th ROI that were imaged at time *t*, and *N*_*it*_ the number of pixels in this set. In cases in which no pixels of a given ROI are imaged in a given frame, a not-a-number (numpy.NaN) value is recorded in place of that ROI's signal at that time.

### 3.5. Requirements and dependencies

The SIMA package and ROI Buddy GUI depend only upon freely available open source software. In particular, the NumPy and SciPy packages (Jones et al., 2001; Oliphant, 2007) for numerical and scientific computing are relied upon heavily throughout. The extraction functionality uses Matplotlib (Hunter, 2007) to generate verification images. The Shapely Python package is used for geometric calculations relating to polygon ROIs. Automated segmentation relies upon Scikit-image (van der Walt et al., 2014) and the Open Source Computer Vision Library (OpenCV), the latter which is also used for ROI registration. The ROI Buddy user interface uses guiqwt (http://code.google.com/p/guiqwt/). HDF5 files are manipulated with the h5py interface (http://www.h5py.org/). These packages are available with a standard scientific Python installation. Since the libtiff C library and its Python bindings enable more memory-efficient handling of multi-page TIFF files, their installation is strongly recommended if SIMA is to be used with large TIFF files containing many frames.

## 4. Discussion and future developments

As a freely available open source software package, SIMA provides a variety of tools to facilitate common steps of dynamic fluorescence imaging analysis, including correction of motion artifacts, segmentation of the field of view into ROIs, and extraction of the fluorescence time-series for each ROI. Data can be imported or exported at various stages of processing with SIMA, so that the package can be used for all stages of analysis, or for any combination of the motion correction, segmentation, and signal extraction. The SIMA package can thus be used flexibly in conjunction with other analysis software. We have thoroughly documented the SIMA package to facilitate use and future collaborative development of this open source project (project hosted on GitHub at https://github.com/losonczylab/sima).

Some of the functionality contained in the SIMA package complements other existing fluorescence imaging acquisition and analysis tools, such as Micro-Manager (Edelstein et al., 2010) and ACQ4 (Campagnola et al., 2014). The TurboReg plug-in for ImageJ (Thevenaz et al., 1998) is capable of correcting motion artifacts that produce mis-aligned frames, but does not allow for correction of within-frame motion artifacts that occur during laser scanning microscopy. The normalized cuts approach to segmentation (Shi and Malik, 2000) is a novel technique for the segmentation of dynamic fluorescence imaging data and is complementary to existing approaches, such as spatio-temporal independent complement analysis (Mukamel et al., 2009), convolutional sparse block coding (Pachitariu et al., 2013), and other methods implemented in ImageJ or CalTracer (http://www.columbia.edu/cu/biology/faculty/yuste/methods.html). In addition to providing this additional approach to segmentation, we have also created a graphical user interface, ROI Buddy, for manual editing of automatically generated ROIs, and for automated registration of ROIs across multiple datasets. ImageJ also provides the ability to draw ROIs and extract signals from image timeseries, but lacks the ability to handle missing data. Overall, a major advantage of SIMA is the integration of these various processing stages into a single tool-kit, allowing for seamless execution of the early stages of analysis of time series laser-scanning microscopy data.

We plan to extend the SIMA package, hopefully in collaboration with the neuroinformatics community, so that future versions have greater functionality. A major need is to extend SIMA with additional methods for automated segmentation. Since the optimal segmentation approach is dependent on the neural structures recorded, the imaging conditions, and the goals of the analysis, we have structured the SIMA module such that additional approaches can be easily implemented and applied to ImagingDataset objects. Integration of other existing segmentation approaches into the SIMA package is an area of active development.

A second avenue for future development is to generalize the applicability of the SIMA package to imaging data acquired by methods other than two-dimensional laser scanning microscopy. In particular, we are interested in extending SIMA to work with newer technologies allowing for three-dimensional imaging within a volume of neural tissue. Such technologies include temporal focusing (Schrödel et al., 2013), light sheet imaging (Verveer et al., 2007), light field imaging (Levoy et al., 2006), and resonance scanning in combination with a piezoelectric crystal. The extension of our software to these technologies should support their broader application.

## Author contributions

Patrick Kaifosh, Jeffrey D. Zaremba, and Nathan B. Danielson developed all software. Attila Losonczy supervised the project. All authors wrote the manuscript.

## Funding

This work was supported by a Howard Hughes Medical Institute International Student Research fellowship (Patrick Kaifosh), NIH Training Grant T32 GM 7367-38 (Nathan B. Danielson), a Human Frontiers Science Program Grant, a McKnight Foundation Memory and Cognitive Disorders Award, the Brain and Behavior Research Foundation, and NIMH grant 1R01MH100631-01A1 (Attila Losonczy).

### Conflict of interest statement

The authors declare that the research was conducted in the absence of any commercial or financial relationships that could be construed as a potential conflict of interest.

## Supplementary material

Extensive documentation for the SIMA package and ROI Buddy GUI is available at http://www.losonczylab.org/sima/. Software and source code can be downloaded from the Python Package Index: https://pypi.python.org/pypi/sima. The source code repository is maintained on GitHub: https://github.com/losonczylab/sima.
